# Study of in vitro transcriptional binding effects and noise using constitutive promoters combined with UP element sequences in *Escherichia coli*

**DOI:** 10.1186/s13036-017-0075-2

**Published:** 2017-11-01

**Authors:** Qiang Yan, Stephen S. Fong

**Affiliations:** 1 Department of Chemical and Life Science Engineering, School of Engineering, Virginia Commonwealth University, West Hall, Room 422, 601 West Main Street, P.O. Box 843028, Richmond, VA 23284-3028 USA; 2 Center for the study of Biological Complexity, Virginia Commonwealth University, Richmond, VA USA

**Keywords:** Upstream element sequence, RNAP α subunits, Gene expression noise, Promoter strength, RNA polymerase, Binding association constant

## Abstract

**Background:**

UP elements (upstream element) are DNA sequences upstream of a promoter that interact with the α-subunit of RNA polymerase (RNAP) and can affect transcription by altering the binding RNAP to DNA. However, details of UP element and binding affinity effects on transcriptional strength are unclear.

**Results:**

Here, we investigated the effects of UP element sequences on gene transcription, binding affinity, and gene expression noise. Addition of UP elements resulted in increased gene expression (maximum 95.7-fold increase) and reduced gene expression noise (8.51-fold reduction). Half UP element sequences at the proximal subsite has little effect on transcriptional strength despite increasing binding affinity by 2.28-fold. In vitro binding assays were used to determine dissociation constants (K_d_) and in the in vitro system, the full range of gene expression occurs in a small range of dissociation constants (25 nM < K_d_ < 45 nM) indicating that transcriptional strength is highly sensitive to small changes in binding affinity.

**Conclusions:**

These results demonstrate the utility of UP elements and provide mechanistic insight into the functional relationship between binding affinity and transcription. Given the centrality of gene expression via transcription to biology, additional insight into transcriptional mechanisms can foster both fundamental and applied research. In particular, knowledge of the DNA sequence-specific effects on expression strength can aid in promoter engineering for different organisms and for metabolic engineering to balance pathway fluxes.

**Electronic supplementary material:**

The online version of this article (10.1186/s13036-017-0075-2) contains supplementary material, which is available to authorized users.

## Background

Transcription and translation are ubiquitous biological processes that are critical for all living systems; however, details of these processes have largely remained unknown until recently. For translation, several tools have been developed based upon base-pair binding strength between the ribosome and ribosome binding sites (RBS) to predict translational initiation rate (TIR) [1, 2]. Detailed understanding of transcription has been more difficult to develop and has largely relied on empirical investigations. A number of promoter libraries have been generated, characterized, modeled and applied over the years [3–6]. These studies largely rely on correlating DNA sequence variations with a functional measure of gene expression. Often, knowledge in terms of functional correlations can be applied to fields such as metabolic engineering where information on functional outcomes is sufficient for situations such as balancing pathway fluxes and metabolic burden reduction [7].

The process of gene expression begins with the initiation of transcription in which the sigma subunit of the RNA polymerase recognizes and binds to DNA at a promoter. Then, the RNA polymerase (RNAP) moves/slides along the DNA molecule to elongate/polymerize the RNA product after a conformational changes known as isomerization [8]. The process is terminated when the RNAP reaches the terminator sequence that follows the protein coding sequence of a gene [9].

Bacterial promoters can consist of three RNAP recognition sequence motifs: the −10 element, the −35 element, and the UP element. The −10 and −35 elements are recognized by the σ subunit of RNAP. The UP element, an AT-rich region located upstream of −35 hexamer (−59 to −38) is recognized by the RNAP α-subunit [10–15]. The most studied naturally occurring UP element is associated with the rRNA promoter *rrnB* P1 in *E. coli* [14–16], which has been characterized in vivo using beta-galactosidase activity for promoter activity. In that study, the UP element-*rrnB* P1 promoter was reported to increase overall activity of beta-galactosidase by at least 30-fold from the promoter alone. Another study investigated different combinations of naturally occurring UP elements with various promoter sequences. The overall expression of the reporter protein was increased 1.5- to 90-fold and in vitro transcription was shown to increase without the presence of the transcription factors – only requirement of the α carboxy terminal domain (αCTD) [12]. This same study also proposed a consensus sequence for UP elements containing highly conserved A/T-rich regions. A library of synthetic UP element-*rrnB* P1 promoters were characterized based upon the expression of LacZ in *E. coli*. From the library, 31 functional UP element sequences (transcription from 136- to 326-fold increase) were aligned and a consensus sequence (−59 NNAAA(A/T)(A/T)T(A/T)TTTTNNAAAANNN -38) was obtained based on the the frequency distribution of the UP elements. The consensus sequence contains two binding parts of αCTD: a proximal binding region (A-tract, −44 to −41) and a distal binding region (A-tract, −57 to −54; T-tract, −53 to −47). On the other hand, other studies showed that a half UP element sequence has little effect on gene transcription level [14]. A following study of half UP elements (proximal subsite and distal subsite) investigated the in vivo interaction with αCTD [17]. The authors revealed that the proximal subsite had a higher preference for αCTD than the distal subsite by increasing binding affinity 170-fold and 16-fold compared to the core promoter, respectively. While studies of UP elements and half UP elements have ranged from construction and characterization of libraries to detailed binding assays, there remains a need for a comprehensive study to span the full range of gene expression function with mechanistic detail.

An affiliated concept when studying gene expression is noise (stochastic fluctuation), which is associated with tightly control of gene expression and cellular performance. Gene expression noise is controlled by two factors, intrinsically random events of transcription and mRNA decay (intrinsic noise) and cell-to-cell variations in regulators, polymerases, and other global factors (extrinsic noise). Recent investigations allow the quantification of protein (GFP) levels in living cells by flow cytometry or fluorescence microscopy. Gene expression noise was calculated as the coefficient of variation (CV), the ratio of the standard deviation to the mean of the population. Extrinsic noise can be predicted and controlled by keeping cell growth at the same phase, as a result, reduction of variability in those factors affecting gene transcription [18]. Although intrinsic noise is more complicated to be predicted due to its nature controlled by both transcription and translation, studies of predicting and controlling intrinsic noise can also be tractable. In *Bacillus subtilis*, it was found that prokaryotic transcription is the dominant source of noise in protein levels, as predicted by basic models of stochastic gene expression [19–21]. Thus, control of promoter fluctuations [22, 23] and gene copy number [24] were reported to lower the intrinsic noise in gene expression. In studying variations in transcription control sequences (promoters and UP elements), changes in intrinsic noise can also be determined along with functional and mechanistic details.

In this study, we seek to add mechanistic details associated with transcription to complement previous work in constructing and characterizing libraries of transcriptional elements. Specifically, this study focuses on the relationship between binding strength (between RNAP and DNA) and functional gene expression strength by determining binding constants for transcriptional control elements (promoter and UP element combinations) with varying binding sites (core promoter, half UP element-core promoter, and UP element-core promoter). Experiments measured promoter strength (using a GFP reporter), binding affinity, and noise for DNA constructs using UP element or half UP element sequences upstream of 19 *E. coli* constitutive core promoters.

## Methods

### Strains, plasmids and culture conditions

*Escherichia coli* NEB10β was used to clone and express all DNA constructs. Plasmid pJ251-GERC (Addgene plasmid #47441) was used for all the promoter-GFP fusion constructs. All *E. coli* recombinant strains were grown in SOC or LB media with 40 μg/mL kanamycin at 30 °C and 230 rpm.

### DNA sequences

A library of 19 constitutive promoters in *Escherichia coli* (BBa_J23100-BBa_J23119) from the Registry of Standard Biological Parts were used as baseline promoter sequences (http://parts.igem.org/Promoters/Catalog/Anderson). A synthetic consensus 24-bp A-T rich UP element sequence (5′-ggaaaattttttttaAAAAAAAAC-3′) was used in this study. A corresponding half UP element sequence (5′-atttgctgctcgtAAAAAAAAAAC-3′) was designed in this study. Eight oligonucleotides were assembled as one core promoter or element-core promoter construct, containing a UNSX (Unique Nucleotide Sequence X) [25], core promoter or UP element-core promoter, synthetic RBS, and 40-bp GFP overlap (Additional file 1: Figure S1).

Polymerase Chain Assembly (PCA) of core promoters and UP element-core promoter sequences was performed in an iCycler thermocycler (Biorad, USA). Oligonucleotide sequences for the PCA reaction can be found in Additional file 1: Table S1. All reactions were conducted in 50 μL volumes containing: 500 nM primers, 10 nM 8 oligonucleotides and 25 μL Q5 Hotstart High-Fidelity 2X Master Mix (New England Biolabs, USA). The thermocycle protocol was as follows: a cycle of 30 s pre-denaturation at 98 °C; 30 cycles of denaturation of 98 °C for 10 s, annealing at 60 °C for 20 s, extension of 25 s at 72 °C; a final cycle extension at 72 °C for 2 min; the reaction system was kept at 4 °C. All reaction products were confirmed on 1% agarose gels. PCA products were extracted using a Zymoclean™ Gel DNA recovery kit (Zymo Research, USA) and measured by Nanodrop 2000c spectrophotometer (Thermo Scientific, USA).

### Plasmid construction

The pJ251-GERC backbone was PCR-amplified from plasmid pJ251-GERC with primer pairs UNSX-f/UNS5-f (Additional file 1: Table S2). The gene fragment with a GFP gene and the Unique Nucleotide Sequence 5 (UNS5) [25] was obtained from IDT (Additional file 1: Table S2). The UP element-core promoter fragment, pJ251-GERC backbone, and GFP fragment were assembled into a recombinant plasmid using NEB Gibson Assembly®2X Master Mix (New England Biolabs, USA) [26]. The assembly product (1 μL) was transformed into *E. coli* NEB10β using electroporation at 3.2 kV for 5 ms using a MicroPulser™ (BioRad, USA). The successful transformed *E. coli* was confirmed by colony PCR reactions (UNSX-f/UNS5-f). The inserted sequence (UP element-core promoter and reporter gene) was validated by sequencing (UNSX-f/UNS5-f) (Eurofins Genomic, USA). Six half UP element-core promoter sequences (J23119, J23102, J23108, J23115, J23109, and J23112) were PCR-amplified from each corresponding UP element-core promoter plasmid template (primers see Additional file 1: Table S2).

### Measurement of GFP fluorescence intensity and gene expression noise

The GFP fluorescence intensity of the cells harvested from liquid cell cultures was measured using flow cytometry. Briefly, an inoculum of *E. coli* strain from agar plate was inoculated into a test tube containing 3 mL LB medium. The seed culture was grown for 12 h and 1 mL of the seed culture was transferred to a secondary culture containing 10 mL LB medium in an Erlenmeyer flask. Cells were collected during exponential growth OD_600_ around 0.4 (about 2.5 h) and stored on ice for at least 1 h to stop cell growth. Cell pellets were then re-suspended with phosphate-buffered saline (PBS) to an optical density between 0.1 and 0.2 at 600 nm using a 1.5 mL centrifuge tube. Flow cytometry analysis was performed using BD Accuri™ C6 flow cytometer (BD Bioscience, USA) by measuring fluorescence from the GFP reporter (excitation wavelength = 488 nm, emission wavelength = 533 nm).

Promoter strength was calculated as a mean fluorescence intensity value of overall cell population. Relative fluorescence intensity was determined by normalized each promoter construct to the lowest promoter J23109 construct. In a single run, gene expression noise can be measured by the distribution of fluorescence intensity of each single cell to the overall cell population, namely, CV (coefficient of variation). CV is defined as the ratio of the standard deviation to the mean of the population. Each construct fluorescence intensity and gene expression noise was tested at least three times. An overall mean value and standard deviation was calculated and presented.

### Electrophoretic mobility shift assay (EMSA)

Electrophoretic mobility shift assays (EMSAs) were performed according to procedures described by Hellman and Fried (2007) with some modifications [27]. A total 20 μL binding reaction included purified *E. coli* RNAP saturated with σ^70^ (New England Biolabs, USA) at different concentrations (0, 10, 21, 42, 63, 85, 170 nM), linearized DNA containing UP element-core promoter/promoter region (10 nM) and a 5X binding buffer (Molecular Probes™, ThermoFisher Scientific, USA). The linearized DNA sequences for EMSAs were PCR-amplified DNA fragments (251 bp) from each pGM serial plasmid using UNSX-f and GFP-r primers. The 5X binding buffer is 750 mM KCl, 0.5 mM dithiothreitol, 0.5 mM EDTA, 50 mM Tris, pH 7.4. The reactions were conducted for 60 min at 37 °C to allow reactions to reach equilibrium. 2 μL EMSA gel-loading solution was added into each 10 μL reaction mixture and loaded onto a pre-electrophoresis, 12% non-denaturing polyacrylamide gel. The gel was run for 35 min at 200 V in 1X TAE buffer. Each gel was stained with SYBR Green (Molecular Probes™, ThermoFisher Scientific, USA) for 20 min, washed with water and visualized using a Gel Doc™ XR+ gel documentation system (BioRad, USA). Gel images were analyzed using ImageLab to quantify fluorescence intensity of each band and determine the fraction of bound DNA. A simplified model equation described below was used to calculate the dissociation constant (K_d_) [28].

$$ \mathrm{B}=\frac{{\mathrm{B}}_{\mathrm{max}}\times \left[\mathrm{RNAP}\right]}{{\mathrm{K}}_{\mathrm{d}}+\left[\mathrm{RNAP}\right]} $$

B is the fraction of DNA that is bound by RNAP, B_max_ is the maximum binding. K_d_ is the dissociation constant. B was plotted versus [RNAP]; K_d_ and B_max_ were calculated using non-linear regression (R^2^ ≥ 0.9).

## Results

### Construction of UP element-core promoter library

**Fig. 1:**
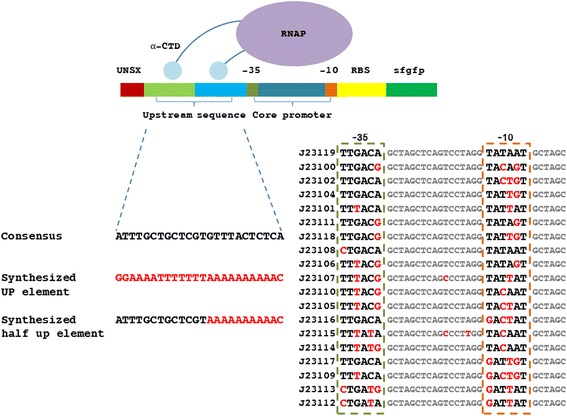
Schematic overview of promoter, UP element-promoter and half UP element-promoter construct designs based on 19 constitutive promoters in *E. coli*. RNAP binds with −35 (marked in dark green) and −10 (marked in orange) in the constitutive promoter to initiate transcription; a full synthesized UP element (sequence in red), a half synthetic UP element (sequence half in red and half in black) and a consensus upstream sequence (sequence in black) located upstream constitutive promoters provides two extra binding sites (marked in green and light blue) with RNAP-αCTD, one binding site, and no binding sites, respectively

Beginning with sequences for 19 constitutive promoters (BBa_J23119 - BBa_J23100) from the Registry of Standard Biological Parts, a library of transcriptional elements with identical genetic context for quantitative testing (using GFP as the reporter). Three types of constructs were generated: 1) promoter alone, 2) promoter with UP element, and 3) promoter with proximal half UP element. The UP element sequence (−59 ggaaaatttttttaAAAAAAAA -38) used in this study was based on the synthetic UP element consensus sequence described previously as −59 NNAAA(A/T)(A/T)T(A/T)TTTTNNAAAANNN -38 [12]. In the same study, the proximal binding region (A-tract, −44 to −41) and the distal binding region (A-tract, −57 to −54; T-tract, −53 to −47) were demonstrated as the most conserved motifs by the frequency distribution of the UP element, while other positions of the UP element sequence tend to have little preference for specific residues. Overall, 40 DNA constructs were assembled for testing, 17 constitutive promoters, 17 promoters with UP element, and 6 half UP element-core promoter constructs (Fig. 1). Two medium strength promoters, J23116 and J23117, were not successfully assembled.

### UP element enhances gene expression level

**Fig. 2:**
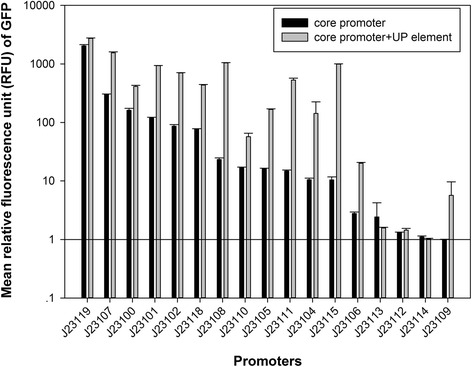
Mean gfp expression value driven by each UP element-promoter or promoter constructs in *E. coli*. The relative fluorescence unit (RFU) of gfp was normalized using the weakest promoter (J23109). Promoter was marked in black bar and UP element-promoter was marked in grey bar. Each constructs was tested at least with three replicates

GFP fluorescence intensity was used as a measurement of expression strength. By conducting measurements during exponential growth, it was assumed that the protein degradation rate was negligible and cell growth rates and GFP maturation rates were similar. We observed no difference in cell growth rate among each construct under same growth condition, and all strains reached OD_600_ of 0.4 at around 2.5 h after inoculation. For each sample, a set cell population (60,000 events) was measured by flow cytometry. Therefore, 17 promoters and 17 UP element-core promoter constructs were tested by measuring fluorescence intensity (Additional file 1: Figure S3 and Additional file 2: Table S3) and calculated relative fluorescence by normalizing each value to the J23109 promoter construct (weakest promoter)**,** shown in Fig. 2.

As a result, 15 out of 17 UP element-core promoter constructs increased their relative fluorescence unit (RFU) compared to the corresponding core promoter alone. The 2 exceptions were the UP element-J23113 construct that had 65% relative fluorescence compared to J23113 and the UP element-J23114 construct that had 93% relative fluorescence compared to J23114. The increase in relative fluorescence ranged from 1.11- to 95.46-fold. The J23119 promoter is the strongest reported wild-type constitutive promoter in *E. coli* and the addition of an UP element upstream of the J23119 core promoter increased RFU 1.34-fold. Among the medium-high strength promoters (RFU > 50, 5 core promoters), the addition of an UP element to the promoter increased RFU 1.76- to 8.13-fold. When investigating the medium-low strength promoters (10 < RFU < 50, 6 core promoters), adding an UP element to the promoter increased fluorescence 3.43- to 44.72-fold. Low strength promoters (RFU < 10, 5 core promoters) showed the broadest range of effects after adding UP elements with increased RFU ranging from 1.11- to 95.46-fold. Generally, inserting a consensus UP element sequence resulted in increase in fluorescence. The addition of an UP element sequence resulted in larger functional changes when added to medium-low strength core promoters than low strength core promoters. For all 6 medium-low strength core promoters, the addition of an UP element sequence increased fluorescence to fall into either medium-high strength promoter or high-strength promoter range (J23108-UP and J23115-UP).

### Effect of half UP element on transcription

Increased gene expression was generally observed when an UP element was added upstream of a constitutive promoter. The added UP element sequence provides two extra binding sites for the αCTD of RNAP to interact with, thus potentially affecting the binding affinity between RNAP and DNA. To further investigate the relationship between binding and transcriptional function, a half UP element consensus sequence at the proximal subsite was designed that would provide one additional binding site (instead of two for a full UP element) for interaction with the RNAP αCTD. Based on previous studies [17], we chose the proximal subsite rather than the distal subsite for three reasons: 1) the consensus proximal subsite is more likely to cause measurable functional changes than the consensus distal subsite; 2) the consensus proximal subsite can potentially stimulate transcription containing only a single copy of αCTD; 3) αCTD preferentially interacts with the proximal subsite region rather than the distal subsite region. Accordingly, the full UP element sequence (−59 ggaaaatttttttaAAAAAAAA -38) was swapped by disrupting the distal conserved sequence (A-tract, −57 to −54; T-tract, −53 to −47) and by remaining the proximal conserved sequence (positions −46 to −38), yielding the half UP element sequence (−59 atttgctgctcgtAAAAAAAAA -38).

Six constitutive promoters were selected including one high-strength promoter (J23119), one medium-high strength promoter (J23102), two medium-low strength promoters (J23108 and J23115), and two low strength promoters (J23112 and J23109). Small increases in fluorescence were observed only for the three constructs using the weakest core promoters (J23115, J23112, J23109) when testing the proximal half UP element constructs (Table 1). For the high strength promoter J23119, inserting a half UP element sequence resulted in a loss of 97% of its original fluorescence intensity, making it function in the range of a medium-low strength promoter (RFU < 50). For the medium-high strength promoter J23102, inserting a half UP element sequence did not change its original strength (50 < RFU < 500). For those medium-low strength promoters, J23108 and J23115, adding half UP element sequences also resulted in no appreciable change in fluorescence intensity (slight decrease for J23108, slight increase for J23115). However, for those low strength promoters, J23112 and J23109, adding half UP element sequences increased fluorescence intensity by 3.81- and 9.93-fold, respectively (full UP element constructs increased expression by 1.11- and 5.68-fold, respectively).

**Table 1:** An electrophoretic mobility shift assay (EMSA) was used to calculate dissociation/association constant for various strength core promoter constructs, half UP element-core promoter constructs, and UP element constructs-core promoter

	RFU	B_max_	K_d_ (nM)	CV	R^2^
J23119	2038.92 ± 104.29	0.99	37.78	68.0 ± 2.98	0.90
J23119 + 1/2UP	44.7 ± 9.41	0.99	21.03	477.8 ± 63.27	0.90
J23119 + UP	2726.88 ± 53.52	0.99	42.02	72.6 ± 1.40	0.90
J23102	86.67 ± 5.87	0.98	47.38	145.09 ± 7.95	0.90
J23102 + 1/2UP	85.37 ± 6.03	0.99	45.51	148.90 ± 9.42	0.90
J23102 + UP	705 ± 4.86	0.99	44.82	92.25 ± 38.40	0.90
J23108	23.31 ± 1.49	0.99	50.06	72.6 ± 7.40	0.92
J23108 + 1/2 UP	18.2 ± 0.30	0.26	21.97	69.1 ± 2.84	0.96
J23108 + UP	1042.43 ± 12.12	0.99	31.43	57.2 ± 0.91	0.90
J23115	10.42 ± 1.26	0.99	55.00	93.3 ± 45.08	0.90
J23115 + 1/2 UP	12.8 ± 0.28	0.16	51.71	71.3 ± 8.96	0.90
J23115 + UP	994.27 ± 13.14	0.99	38.14	64.54 ± 5.71	0.90
J23112	1.30 ± 0.040	0.15	50.8	321.96 ± 21.96	0.90
J23112 + 1/2UP	4.96 ± 0.10	0.64	68.16	440.39 ± 19.24	0.91
J23112 + UP	1.44 ± 0.11	0.56	12.55	296.93 ± 24.64	0.90
J23109	1 ± 0.005	0.16	46.05	258.17 ± 111.29	0.90
J23109 + 1/2UP	9.93 ± 0.66	0.66	64.75	171.87 ± 31.28	0.90
J23109 + UP	5.68 ± 0.99	0.50	7.44	158.75 ± 28.40	0.93

### Binding affinity

Given the variation in results using full UP element sequences and half UP element sequences, a series of in vitro binding assays were conducted to determine binding constants for the interaction of RNAP with the transcriptional control sequences to gain better mechanistic insight. Electrophoretic mobility shift assays (EMSAs) using the *E. coli* holoenzyme saturated with σ^70^ and DNA (251-bp double-stranded DNA containing the core promoter or half UP element and UP element constructs) were used to measure binding affinity. For interaction to occur between the αCTD of RNAP and the binding sites of an UP element, it is necessary to add the σ^70^ subunit to the RNAP holoenzyme. By using a short 251-bp double-stranded DNA sequence non-specific binding is negligible and the binding affinity (K_a_) differences should reflect the binding affinity discrepancy between each core promoter or UP element-core promoter and RNAP. EMSAs were conducted for 18 DNA constructs (6 promoters, 6 half UP element-core promoter sequences, and 6 UP element-core promoter sequences) (representative figures see Additional file 1: Figure S4).

From the EMSA data, dissociation constants (K_d_) were calculated using an equation to model binding affinity (see Methods) and dissociation constants for the different DNA constructs ranged from 7.44 nM – 68.16 nM (Table 1). Superficially, the expectation was that the addition of more potential binding sites (one site for half UP element and two sites for full UP element) should lead to increased binding affinity (lower K_d_). In most cases the addition of a half UP element or a full UP element to a promoter did lead to increased binding affinity/lower K_d_.

However, the relationship between addition of a half UP element or full UP element to fluorescence intensity was not as clear. For the high strength promoter (J23119), adding a consensus UP element sequence produced a slight decrease in binding affinity (higher K_d_) and increase in fluorescence intensity. Addition of a half UP element sequence to J23119 resulted in a significant increase in binding affinity (1.80-fold) and a 45.6-fold decrease in fluorescence intensity. For the medium-high strength promoter J23102, inserting an UP element or half UP element produced small increases in binding affinity (slightly lower K_d_), and interestingly the small change in binding upon adding an UP element (K_d_ = 47.38 nM to K_d_ = 44.82 nM) resulted in an 8.13-fold increase in fluorescence intensity. For the two medium-low strength core promoters (J23108 and J23115), addition of an UP element increased the promoter binding affinity by 1.59- and 1.44-fold, respectively, and relative fluorescence for both constructs also increased (44.72- and 95.42-fold, respectively). The two low-strength promoters (J23112 and J23109) showed increased binding affinity when an UP element was added, but relative fluorescence did not change appreciably.

**Fig. 3:**
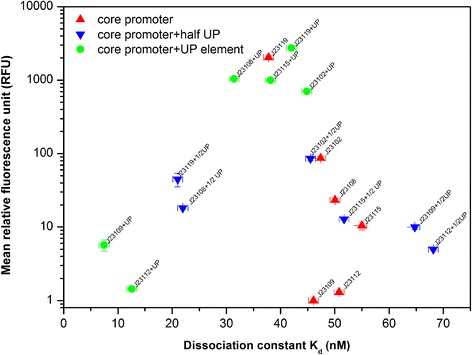
An overall distribution of promoter strength (RFU) against promoter-RNAP binding affinity (dissociation constant, Kd) using 6 promoter and their corresponding promoter-half UP element and promoter-UP element construct. Promoter construct was marked as triangle in red, promoter-half UP element construct was marked as inverted triangle in blue, and promoter-UP element construct was marked as circle in green

The relationship between binding strength and transcriptional function was analyzed by plotting RFU against dissociation constant (K_d_), shown in Fig. 3. Both strong binding affinity (K_d_ < 25 nM) and weak binding affinity (K_d_ > 45 nM) resulted in low RFU. The dynamic range of functional expression was empirically found to be a relatively small range of moderate binding affinity (25 nM < K_d_ < 45 nM). It appears that transcriptional strength is highly sensitive to small changes in binding affinity and that functional expression varies in a small binding range. This would be consistent with the transcriptional mechanism where binding between a promoter and RNAP that is too strong would hinder RNAP movement for mRNA elongation, while binding that is too weak would lead to a low probability of transcription initiation [29, 30].

### Gene expression noise with UP elements

Gene expression noise was examined using the coefficient variation (CV) for fluorescence measurements of the 17 constitutive promoters and the 17 UP element-core promoter constructs. The CV value is defined as the standard deviation over the mean fluorescence intensity, which reflects the distribution of fluorescence intensity for each construct (intrinsic noise and extrinsic noise). Given that the cell growth conditions (medium, temperature, shaking speed, and growth phase-exponential phase) were identical for all measurements, the CV likely is a reflection of intrinsic noise associated with the transcriptional behavior of each tested construct.

Overall, CV values decreased when an UP element sequence was added to a constitutive promoter, shown in Additional file 2: Table S4. For medium-high strength promoters (RFU > 50), four out of five promoters showed a decrease in CV (20% - 50% decrease) after adding an UP element sequence. For medium-low strength promoters (10 < RFU < 50), all promoters (six of six) showed a decrease in CV (21% - 88% decrease) after adding an UP element sequence. All low strength promoters (RFU < 10), (five out of five) also showed decreases in CV (7% - 71% decrease) after adding UP element sequences. Interestingly, we found that the effect of UP elements on noise appeared to be greatest for lower strength constructs.

**Fig. 4:**
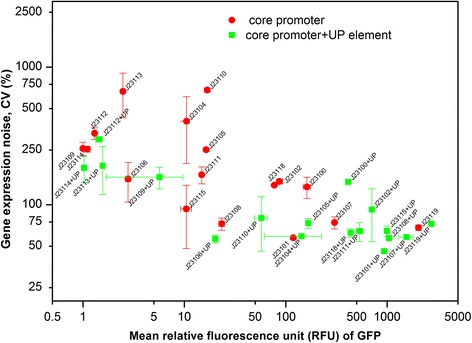
An overall distribution of gene expression noise (CV value) against promoter strength (RFU) using promoter and UP element-promoter constructs in *E. coli*. Promoter was marked as circle in red and promoter UP-element was marked as square in green

A plot was generated to consider correlations between promoter strength (RFU) and gene expression noise (Fig. 4). Transcriptional constructs containing an UP element tended to have higher functional strength and lower noise (CV) than constitutive promoters alone. At lower fluorescence intensity, the expression noise increased due to the weak control of the promoter (low signal to noise ratio). As fluorescence intensity increased (RFU > 15), the CV value decreased indicating a reduction in noise.

## Discussion

In this study, synthetic UP element sequences (full and half UP elements) were used in conjunction with constitutive promoters to study relationships between binding, noise, and functional expression. A total of 40 DNA constructs were tested and 18 constructs were used for detailed characterization of fluorescence intensity, binding affinity, and expression noise. Results demonstrated that: 1) UP elements can significantly increase expression of a gene, but half-UP elements have minimal effects, 2) functional gene expression occurs in a small range of binding affinity, and 3) gene expression noise can be reduced by the addition of UP element sequences.

Based on a translation level measurement of promoter strength, our results showed that combining a UP element to a core promoter sequence was capable of increasing transcription levels regardless of the promoter strength (Fig. 2), sometimes even producing an almost 100-fold increase in function (95.7-fold increase when UP element was added to the J23115 promoter). These findings are consistent with previous studies that showed 1.5- to 90-fold increases in expression using UP elements with *E. coli* RNA ribosome promoter (*rrnD* P1 and *rrnB* P2), phage promoter (lambda P_R_), and a hybrid Lac promoter [31]. The fact that the addition of half UP element sequences showed little effect on fluorescence intensity was also in consistence with previous studies [14].

In this study, by generating a distribution of constructs with varying binding affinity and expression strength, it was empirically found that the full range of functional gene expression occurs in small range of moderate binding affinity (25 nM < K_d_ < 45 nM). Weak binding affinity can result in a failure of transcription initiation due to difficulty in recognition of promoter region; too strong binding affinity can potentially adversely affect the second step of transcription where a conformational changes (isomerization) of the RNAP-promoter complex to facilitate elongation, which is reported to generally be a rate-limiting step [8, 32]. These findings can provide a molecular level mechanism that is helpful to explain some of the previously unclear fluorescence intensity results regarding the mechanistic contributions of UP elements and half UP elements contributing to transcription. Previous studies either observed that half UP element does not change gene expression level [14] or demonstrated that a proximal half UP element sequence can enhance RNAP binding affinity to promoter [17]. In the present study, for all the functional constitutive promoters used with a half UP element (J23119, J23102, J23108, and J23115), the addition of the half UP element increased binding affinity (lower K_d_), however, the fluorescence intensity dropped drastically. Given the small range of binding affinity for expression (25 nM < K_d_ < 45 nM) and the shape of the function (Fig. 3), the changes in fluorescence intensity for the half UP element constructs become clearer.

The experimental characterization of promoter strength is an essential baseline in computational aided promoter design since the experimental values can be set as an input and restriction setup in the initial model construction [33, 34]. These thermodynamic transcriptional models are constructed based on a gene expression dataset of promoter libraries with the information of promoters’ base-pair variations; subsequently, a predicted promoter binding energy values at each promoter’s base-pair position (from −1 to −40) are calculated to form an initial model; finally, the model can be further improved with cycles of design-build-test. The total 40 promoters database (promoter strength, dissociation constant, gene expression noise) obtained in this study can be a useful guide for a model-based promoter design: First, the current well-developed models focus on mainly the conserved core promoter motifs (−10 and −35). The experimental data obtained from this study can extend to the third binding motif, UP element sequence. Second, our observations that the functional promoter dissociation constant fell into a certain range can be a valuable restriction conditions to optimize a model. Furthermore, there are few models built for the simulation of gene expression noise due to unpredictable nature of transcriptional initiation. Our results may provide a valuable database for understanding the probabilistic natural behavior.

Other studies have tested different methods to control gene expression noise at the transcriptional level using methods such as miRNAs [35] and predicting and tuning promoter strength [36, 37]. In this study, it was found that gene expression noise is reduced when inserting an UP element upstream of a promoter. By controlling the cell culture condition (medium, inoculation, temperature, pH, aeration), variation in gene expression levels for each constructs within a population should be dominated mainly by transcription due to pulses of messenger RNA produced in a probabilistic manner [38]. One explanation is that by UP element can increase the binding affinity between promoter and RNAP, therefore, such improvement can help provide tight control of GFP mRNA levels. This is possibly analogous to microRNA research that has demonstrated reduced protein expression noise [39, 40]. Generally, it was found that constructs with relative fluorescence above 15 RFU showed greatly reduced gene expression noise. A similar correlation between gene noise and expression mean value has been reported in *Saccharomyces cerevisiae* [41]. One explanation could be that an increase of promoter activity would change the frequency of transcriptional burst.

Recently, the transcription-based promoter approach has been demonstrated a successful modular optimization strategy to improve the production of many chemicals [42–44]. Yet, there is still a need for obtaining well-characterized libraries of promoters in *E. coli*. Recently, Zhou et al. characterized 104 native promoter-5′-UTR complexes (PUTR) based on mRNA-seq data. The strength of the 104 PUTRs ranged from 1- to 1370-fold in the translation level [45]. In this study, the strength of our updated promoter libraries varied a larger range (from 1-fold to 2734-fold) compared to the study. Specifically, based on both promoter strength and gene expression noise, we proposed four optimal UP element-core promoters that can contribute to future synthetic biology and metabolic engineering applications: a high strength promoter (J23119 + UP), medium-high strength promoter (J23101 + UP), medium-low strength promoter (J23106 + UP), and low strength promoter (J23109 + UP). Such well-characterized libraries have the potential to contribute to metabolic engineering for pathway balancing, promoter engineering and metabolic burden reduction [7].

## Conclusion

In this study, synthetic UP element sequences (full and half UP elements) with constitutive promoters were investigated the relationships between binding, noise, and functional expression. A total of 40 DNA constructs were tested and 18 constructs were used for detailed characterization of fluorescence intensity, binding affinity, and expression noise. Our results showed that UP element proves to increase gene expression level; however, half-UP elements have little effects. Moreover, decent gene expression level occurs in a small range of binding affinity. UP element can facilitate to reduce gene expression noise. Hence, our observations demonstrate the utility of UP elements and provide mechanistic perspective into the functional relationship between binding affinity and gene expression.

## Additional files

Additional file 1: Figure S1. Schematic graph of assembly 8 oligonucleotides to a 251 bp dsDNA by polymerase chain assembly. **Figure S2.** Effect of annealing temperature on PCA the 8 oligonucleotides. **Table S1.** Oligonucleotide information for polymerase chain assembly reaction (PCA). **Table S2.** Primers and DNA sequences used in this study. (DOCX 341 kb)

Additional file 2: Table S3. Raw experimental data for fluorescence unit and variance of coefficient. **Table S4**. Raw data for EMSA to calculate dissociation constant (K_d_) and maximum binding fraction (Y_max_). (XLSX 22 kb)

## Abbreviations

CV: Coefficient of variance
GFP: Green fluorescence protein
PUTR: Promoter-5′-UTR complexes
RBS: Ribosome binding site
RFU: Relative fluorescence unit
RNAP: RNA polymerase
TIR: Translational initiation rate
αCTD: α-carboxy terminal domain
