# Supplementary material for: Study of in vitro transcriptional binding effects and noise using constitutive promoters combined with UP element sequences in Escherichia coli
Source: J Biol Eng. 2017 Nov 1;11:33. doi: 10.1186/s13036-017-0075-2 (PMC5664571; doi:10.1186/s13036-017-0075-2)
Supplement: Supplementary file 1 — Schematic graph of assembly 8 oligonucleotides to a 251 bp dsDNA by polymerase chain assembly. Figure S2. Effect of annealing temperature on PCA the 8 oligonucleotides. Table S1. Oligonucleotide information for polymerase chain assembly reaction (PCA). Table S2. Primers and DNA sequences used in this study. (DOCX 341 kb) [file 13036_2017_75_MOESM1_ESM.docx]

**Additional file 1**

**Title**: study of *in vitro* transcriptional binding effects and noise using standard constitutive promoters combined with UP element sequences in *Escherichia coli*

Authors and affiliations

Qiang Yan^1^ and Stephen S Fong^1,2*^

^1^Department of Chemical and Life Science Engineering, Virginia Commonwealth University, Richmond, Virginia

^2^Center for the study of Biological Complexity, Virginia Commonwealth University, Richmond, Virginia

*Dr. Stephen Fong, Virginia Commonwealth University, School of Engineering, Department of Chemical and Life Science Engineering West Hall, Room 422, 601 West Main Street, P.O. Box 843028, Richmond, Virginia 23284-3028; [ssfong@vcu.edu](mailto:ssfong@vcu.edu); (804)827-7038

**Running title:** Study of UP element binding effects in *E. coli*

**Keywords**: upstream element sequence, RNAP α subunits, gene expression noise, promoter strength, RNA polymerase, binding association constant


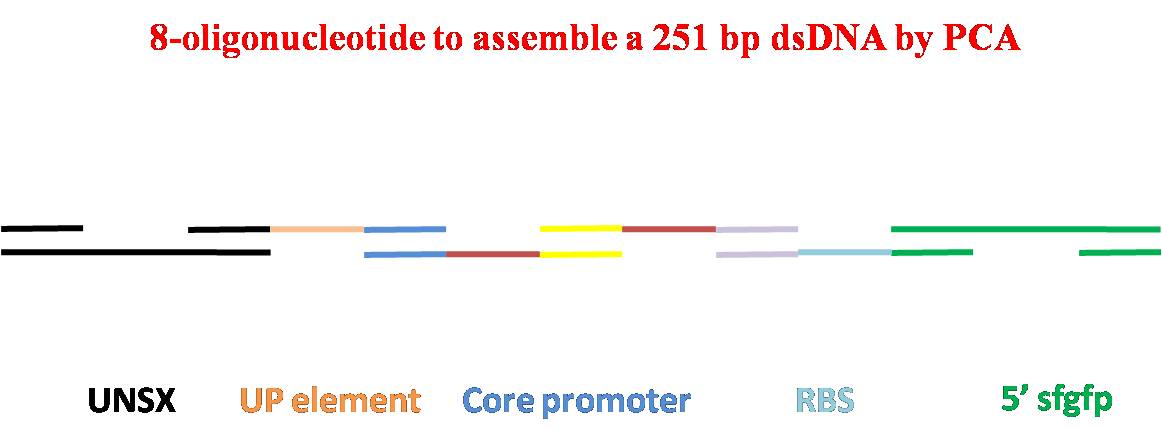


**Fig. S1.** Schematic graph of assembly 8 oligonucleotides to a 251 bp dsDNA by polymerase chain assembly.


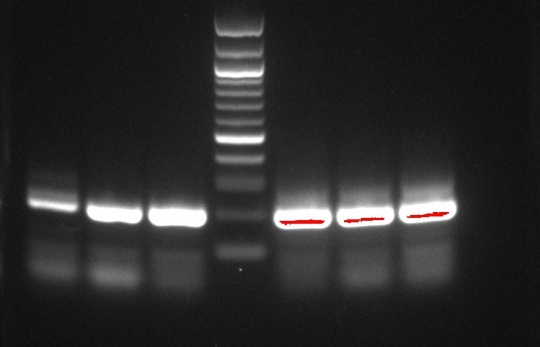


**Fig. S2.** Effect of annealing temperature on PCA the 8 oligonucleotides. From left to right lanes are assembly products at annealing temperature 50, 55, 60, 1kb ladder, 65, 70, 72°C.

**Table S1** Oligo nucleotide information for polymerase chain assembly reaction (PCA)

| Promoter | Oligo name | Sequence |
| --- | --- | --- |
| J23119 | oGM196 | 5- CCA GGA TAC ATA GAT TAC CAC AAC TCC GAG -3 |
|  | oGM347 | 5- ACG AGC AGC AAA TAT AGG TGG TGG AAG GGC TCG GAG TTG TGG TAA TCT ATG TAT CCT GG -3 |
|  | oGM52 | 5- ACC TAT ATT TGC TGC TCG TGT TTA CTC TCA TTG ACA GCT AGC TCA GTC CTA GGT ATA ATG -3 |
|  | oGM53 | 5- ACT CAT CAG ACC GGA AAG CAC ATC CGG TGA CAG CTG CTA GCA TTA TAC CTA GGA CTG AGC -3 |
|  | oGM54 | 5- TGC TTT CCG GTC TGA TGA GTC CGT GAG GAC GAA ACA GCC TCT ACA AAT AAT TTT GTT TAA -3 |
|  | oGM335 | 5- AGG TAA TTC TTT CCT TAT AAA CTA TTG GAC TTA TTA AAC AAA ATT ATT TGT AGA GGC T -3 |
|  | oGM343 | 5- GTT TAT AAG GAA AGA ATT ACC TAT GCG TAA AGG CGA AGA GCT GTT CAC TGG TGT CGT CCC -3 |
|  | oGM345 | 5- GGG ACG ACA CCA GTG AAC AGC -3 |
| J23119+UP | oGM196 | 5- CCA GGA TAC ATA GAT TAC CAC AAC TCC GAG -3 |
|  | oGM198 | 5- TAA AAA AAT TTT CCA TAG GTG GTG GAA GGG CTC GGA GTT GTG GTA ATC TAT GTA TCC TGG -3 |
|  | oGM58 | 5- ACC TAT GGA AAA TTT TTT TAA AAA AAA AAC TTG ACA GCT AGC TCA GTC CTA GGT ATA ATG -3 |
|  | oGM53 | 5- ACT CAT CAG ACC GGA AAG CAC ATC CGG TGA CAG CTG CTA GCA TTA TAC CTA GGA CTG AGC -3 |
|  | oGM54 | 5- TGC TTT CCG GTC TGA TGA GTC CGT GAG GAC GAA ACA GCC TCT ACA AAT AAT TTT GTT TAA -3 |
|  | oGM335 | 5- AGG TAA TTC TTT CCT TAT AAA CTA TTG GAC TTA TTA AAC AAA ATT ATT TGT AGA GGC T -3 |
|  | oGM343 | 5- GTT TAT AAG GAA AGA ATT ACC TAT GCG TAA AGG CGA AGA GCT GTT CAC TGG TGT CGT CCC -3 |
|  | oGM345 | 5- GGG ACG ACA CCA GTG AAC AGC -3 |
| J23100 | oGM196 | 5- CCA GGA TAC ATA GAT TAC CAC AAC TCC GAG -3 |
|  | oGM347 | 5- ACG AGC AGC AAA TAT AGG TGG TGG AAG GGC TCG GAG TTG TGG TAA TCT ATG TAT CCT GG -3 |
|  | oGM59 | 5- ACC TAT ATT TGC TGC TCG TGT TTA CTC TCA TTG ACG GCT AGC TCA GTC CTA GGT ACA GTG -3 |
|  | oGM60 | 5- ACT CAT CAG ACC GGA AAG CAC ATC CGG TGA CAG CTG CTA GCA CTG TAC CTA GGA CTG AGC -3 |
|  | oGM54 | 5- TGC TTT CCG GTC TGA TGA GTC CGT GAG GAC GAA ACA GCC TCT ACA AAT AAT TTT GTT TAA -3 |
|  | oGM335 | 5- AGG TAA TTC TTT CCT TAT AAA CTA TTG GAC TTA TTA AAC AAA ATT ATT TGT AGA GGC T -3 |
|  | oGM343 | 5- GTT TAT AAG GAA AGA ATT ACC TAT GCG TAA AGG CGA AGA GCT GTT CAC TGG TGT CGT CCC -3 |
|  | oGM345 | 5- GGG ACG ACA CCA GTG AAC AGC -3 |
| J23100+UP | oGM196 | 5- CCA GGA TAC ATA GAT TAC CAC AAC TCC GAG -3 |
|  | oGM198 | 5- TAA AAA AAT TTT CCA TAG GTG GTG GAA GGG CTC GGA GTT GTG GTA ATC TAT GTA TCC TGG -3 |
|  | oGM61 | 5- ACC TAT GGA AAA TTT TTT TAA AAA AAA AAC TTG ACG GCT AGC TCA GTC CTA GGT ACA GTG -3 |
|  | oGM60 | 5- ACT CAT CAG ACC GGA AAG CAC ATC CGG TGA CAG CTG CTA GCA CTG TAC CTA GGA CTG AGC -3 |
|  | oGM54 | 5- TGC TTT CCG GTC TGA TGA GTC CGT GAG GAC GAA ACA GCC TCT ACA AAT AAT TTT GTT TAA -3 |
|  | oGM335 | 5- AGG TAA TTC TTT CCT TAT AAA CTA TTG GAC TTA TTA AAC AAA ATT ATT TGT AGA GGC T -3 |
|  | oGM343 | 5- GTT TAT AAG GAA AGA ATT ACC TAT GCG TAA AGG CGA AGA GCT GTT CAC TGG TGT CGT CCC -3 |
|  | oGM345 | 5- GGG ACG ACA CCA GTG AAC AGC -3 |
| J23102 | oGM196 | 5- CCA GGA TAC ATA GAT TAC CAC AAC TCC GAG -3 |
|  | oGM347 | 5- ACG AGC AGC AAA TAT AGG TGG TGG AAG GGC TCG GAG TTG TGG TAA TCT ATG TAT CCT GG -3 |
|  | oGM62 | 5- ACC TAT ATT TGC TGC TCG TGT TTA CTC TCA TTG ACA GCT AGC TCA GTC CTA GGT ACT GTG -3 |
|  | oGM63 | 5- ACT CAT CAG ACC GGA AAG CAC ATC CGG TGA CAG CTG CTA GCA CAG TAC CTA GGA CTG AGC -3 |
|  | oGM54 | 5- TGC TTT CCG GTC TGA TGA GTC CGT GAG GAC GAA ACA GCC TCT ACA AAT AAT TTT GTT TAA -3 |
|  | oGM335 | 5- AGG TAA TTC TTT CCT TAT AAA CTA TTG GAC TTA TTA AAC AAA ATT ATT TGT AGA GGC T -3 |
|  | oGM343 | 5- GTT TAT AAG GAA AGA ATT ACC TAT GCG TAA AGG CGA AGA GCT GTT CAC TGG TGT CGT CCC -3 |
|  | oGM345 | 5- GGG ACG ACA CCA GTG AAC AGC -3 |
| J23102+UP | oGM196 | 5- CCA GGA TAC ATA GAT TAC CAC AAC TCC GAG -3 |
|  | oGM198 | 5- TAA AAA AAT TTT CCA TAG GTG GTG GAA GGG CTC GGA GTT GTG GTA ATC TAT GTA TCC TGG -3 |
|  | oGM64 | 5- ACC TAT GGA AAA TTT TTT TAA AAA AAA AAC TTG ACA GCT AGC TCA GTC CTA GGT ACT GTG -3 |
|  | oGM63 | 5- ACT CAT CAG ACC GGA AAG CAC ATC CGG TGA CAG CTG CTA GCA CAG TAC CTA GGA CTG AGC -3 |
|  | oGM54 | 5- TGC TTT CCG GTC TGA TGA GTC CGT GAG GAC GAA ACA GCC TCT ACA AAT AAT TTT GTT TAA -3 |
|  | oGM335 | 5- AGG TAA TTC TTT CCT TAT AAA CTA TTG GAC TTA TTA AAC AAA ATT ATT TGT AGA GGC T -3 |
|  | oGM343 | 5- GTT TAT AAG GAA AGA ATT ACC TAT GCG TAA AGG CGA AGA GCT GTT CAC TGG TGT CGT CCC -3 |
|  | oGM345 | 5- GGG ACG ACA CCA GTG AAC AGC -3 |
| J23104 | oGM196 | 5- CCA GGA TAC ATA GAT TAC CAC AAC TCC GAG -3 |
|  | oGM347 | 5- ACG AGC AGC AAA TAT AGG TGG TGG AAG GGC TCG GAG TTG TGG TAA TCT ATG TAT CCT GG -3 |
|  | oGM65 | 5- ACC TAT ATT TGC TGC TCG TGT TTA CTC TCA TTG ACA GCT AGC TCA GTC CTA GGT ATT GTG -3 |
|  | oGM66 | 5- ACT CAT CAG ACC GGA AAG CAC ATC CGG TGA CAG CTG CTA GCA CAA TAC CTA GGA CTG AGC -3 |
|  | oGM54 | 5- TGC TTT CCG GTC TGA TGA GTC CGT GAG GAC GAA ACA GCC TCT ACA AAT AAT TTT GTT TAA -3 |
|  | oGM335 | 5- AGG TAA TTC TTT CCT TAT AAA CTA TTG GAC TTA TTA AAC AAA ATT ATT TGT AGA GGC T -3 |
|  | oGM343 | 5- GTT TAT AAG GAA AGA ATT ACC TAT GCG TAA AGG CGA AGA GCT GTT CAC TGG TGT CGT CCC -3 |
|  | oGM345 | 5- GGG ACG ACA CCA GTG AAC AGC -3 |
| J23104+UP | oGM196 | 5- CCA GGA TAC ATA GAT TAC CAC AAC TCC GAG -3 |
|  | oGM198 | 5- TAA AAA AAT TTT CCA TAG GTG GTG GAA GGG CTC GGA GTT GTG GTA ATC TAT GTA TCC TGG -3 |
|  | oGM67 | 5- ACC TAT GGA AAA TTT TTT TAA AAA AAA AAC TTG ACA GCT AGC TCA GTC CTA GGT ATT GTG -3 |
|  | oGM66 | 5- ACT CAT CAG ACC GGA AAG CAC ATC CGG TGA CAG CTG CTA GCA CAA TAC CTA GGA CTG AGC -3 |
|  | oGM54 | 5- TGC TTT CCG GTC TGA TGA GTC CGT GAG GAC GAA ACA GCC TCT ACA AAT AAT TTT GTT TAA -3 |
|  | oGM335 | 5- AGG TAA TTC TTT CCT TAT AAA CTA TTG GAC TTA TTA AAC AAA ATT ATT TGT AGA GGC T -3 |
|  | oGM343 | 5- GTT TAT AAG GAA AGA ATT ACC TAT GCG TAA AGG CGA AGA GCT GTT CAC TGG TGT CGT CCC -3 |
|  | oGM345 | 5- GGG ACG ACA CCA GTG AAC AGC -3 |
| J23101 | oGM196 | 5- CCA GGA TAC ATA GAT TAC CAC AAC TCC GAG -3 |
|  | oGM347 | 5- ACG AGC AGC AAA TAT AGG TGG TGG AAG GGC TCG GAG TTG TGG TAA TCT ATG TAT CCT GG -3 |
|  | oGM68 | 5- ACC TAT ATT TGC TGC TCG TGT TTA CTC TCA TTT ACA GCT AGC TCA GTC CTA GGT ATT ATG -3 |
|  | oGM69 | 5- ACT CAT CAG ACC GGA AAG CAC ATC CGG TGA CAG CTG CTA GCA TAA TAC CTA GGA CTG AGC -3 |
|  | oGM54 | 5- TGC TTT CCG GTC TGA TGA GTC CGT GAG GAC GAA ACA GCC TCT ACA AAT AAT TTT GTT TAA -3 |
|  | oGM335 | 5- AGG TAA TTC TTT CCT TAT AAA CTA TTG GAC TTA TTA AAC AAA ATT ATT TGT AGA GGC T -3 |
|  | oGM343 | 5- GTT TAT AAG GAA AGA ATT ACC TAT GCG TAA AGG CGA AGA GCT GTT CAC TGG TGT CGT CCC -3 |
|  | oGM345 | 5- GGG ACG ACA CCA GTG AAC AGC -3 |
| J23101+UP | oGM196 | 5- CCA GGA TAC ATA GAT TAC CAC AAC TCC GAG -3 |
|  | oGM198 | 5- TAA AAA AAT TTT CCA TAG GTG GTG GAA GGG CTC GGA GTT GTG GTA ATC TAT GTA TCC TGG -3 |
|  | oGM70 | 5- ACC TAT GGA AAA TTT TTT TAA AAA AAA AAC TTT ACA GCT AGC TCA GTC CTA GGT ATT ATG -3 |
|  | oGM69 | 5- ACT CAT CAG ACC GGA AAG CAC ATC CGG TGA CAG CTG CTA GCA TAA TAC CTA GGA CTG AGC -3 |
|  | oGM54 | 5- TGC TTT CCG GTC TGA TGA GTC CGT GAG GAC GAA ACA GCC TCT ACA AAT AAT TTT GTT TAA -3 |
|  | oGM335 | 5- AGG TAA TTC TTT CCT TAT AAA CTA TTG GAC TTA TTA AAC AAA ATT ATT TGT AGA GGC T -3 |
|  | oGM343 | 5- GTT TAT AAG GAA AGA ATT ACC TAT GCG TAA AGG CGA AGA GCT GTT CAC TGG TGT CGT CCC -3 |
|  | oGM345 | 5- GGG ACG ACA CCA GTG AAC AGC -3 |
| J23111 | oGM196 | 5- CCA GGA TAC ATA GAT TAC CAC AAC TCC GAG -3 |
|  | oGM347 | 5- ACG AGC AGC AAA TAT AGG TGG TGG AAG GGC TCG GAG TTG TGG TAA TCT ATG TAT CCT GG -3 |
|  | oGM71 | 5- ACC TAT ATT TGC TGC TCG TGT TTA CTC TCA TTG ACG GCT AGC TCA GTC CTA GGT ATA GTG -3 |
|  | oGM72 | 5- ACT CAT CAG ACC GGA AAG CAC ATC CGG TGA CAG CTG CTA GCA CTA TAC CTA GGA CTG AGC -3 |
|  | oGM54 | 5- TGC TTT CCG GTC TGA TGA GTC CGT GAG GAC GAA ACA GCC TCT ACA AAT AAT TTT GTT TAA -3 |
|  | oGM335 | 5- AGG TAA TTC TTT CCT TAT AAA CTA TTG GAC TTA TTA AAC AAA ATT ATT TGT AGA GGC T -3 |
|  | oGM343 | 5- GTT TAT AAG GAA AGA ATT ACC TAT GCG TAA AGG CGA AGA GCT GTT CAC TGG TGT CGT CCC -3 |
|  | oGM345 | 5- GGG ACG ACA CCA GTG AAC AGC -3 |
| J23111+UP | oGM196 | 5- CCA GGA TAC ATA GAT TAC CAC AAC TCC GAG -3 |
|  | oGM198 | 5- TAA AAA AAT TTT CCA TAG GTG GTG GAA GGG CTC GGA GTT GTG GTA ATC TAT GTA TCC TGG -3 |
|  | oGM73 | 5- ACC TAT GGA AAA TTT TTT TAA AAA AAA AAC TTG ACG GCT AGC TCA GTC CTA GGT ATA GTG -3 |
|  | oGM72 | 5- ACT CAT CAG ACC GGA AAG CAC ATC CGG TGA CAG CTG CTA GCA CTA TAC CTA GGA CTG AGC -3 |
|  | oGM54 | 5- TGC TTT CCG GTC TGA TGA GTC CGT GAG GAC GAA ACA GCC TCT ACA AAT AAT TTT GTT TAA -3 |
|  | oGM335 | 5- AGG TAA TTC TTT CCT TAT AAA CTA TTG GAC TTA TTA AAC AAA ATT ATT TGT AGA GGC T -3 |
|  | oGM343 | 5- GTT TAT AAG GAA AGA ATT ACC TAT GCG TAA AGG CGA AGA GCT GTT CAC TGG TGT CGT CCC -3 |
|  | oGM345 | 5- GGG ACG ACA CCA GTG AAC AGC -3 |
| J23118 | oGM196 | 5- CCA GGA TAC ATA GAT TAC CAC AAC TCC GAG -3 |
|  | oGM347 | 5- ACG AGC AGC AAA TAT AGG TGG TGG AAG GGC TCG GAG TTG TGG TAA TCT ATG TAT CCT GG -3 |
|  | oGM74 | 5- ACC TAT ATT TGC TGC TCG TGT TTA CTC TCA TTG ACG GCT AGC TCA GTC CTA GGT ATT GTG -3 |
|  | oGM66 | 5- ACT CAT CAG ACC GGA AAG CAC ATC CGG TGA CAG CTG CTA GCA CAA TAC CTA GGA CTG AGC -3 |
|  | oGM54 | 5- TGC TTT CCG GTC TGA TGA GTC CGT GAG GAC GAA ACA GCC TCT ACA AAT AAT TTT GTT TAA -3 |
|  | oGM335 | 5- AGG TAA TTC TTT CCT TAT AAA CTA TTG GAC TTA TTA AAC AAA ATT ATT TGT AGA GGC T -3 |
|  | oGM343 | 5- GTT TAT AAG GAA AGA ATT ACC TAT GCG TAA AGG CGA AGA GCT GTT CAC TGG TGT CGT CCC -3 |
|  | oGM345 | 5- GGG ACG ACA CCA GTG AAC AGC -3 |
| J23118+UP | oGM196 | 5- CCA GGA TAC ATA GAT TAC CAC AAC TCC GAG -3 |
|  | oGM198 | 5- TAA AAA AAT TTT CCA TAG GTG GTG GAA GGG CTC GGA GTT GTG GTA ATC TAT GTA TCC TGG -3 |
|  | oGM75 | 5- ACC TAT GGA AAA TTT TTT TAA AAA AAA AAC TTG ACG GCT AGC TCA GTC CTA GGT ATT GTG -3 |
|  | oGM66 | 5- ACT CAT CAG ACC GGA AAG CAC ATC CGG TGA CAG CTG CTA GCA CAA TAC CTA GGA CTG AGC -3 |
|  | oGM54 | 5- TGC TTT CCG GTC TGA TGA GTC CGT GAG GAC GAA ACA GCC TCT ACA AAT AAT TTT GTT TAA -3 |
|  | oGM335 | 5- AGG TAA TTC TTT CCT TAT AAA CTA TTG GAC TTA TTA AAC AAA ATT ATT TGT AGA GGC T -3 |
|  | oGM343 | 5- GTT TAT AAG GAA AGA ATT ACC TAT GCG TAA AGG CGA AGA GCT GTT CAC TGG TGT CGT CCC -3 |
|  | oGM345 | 5- GGG ACG ACA CCA GTG AAC AGC -3 |
| J23108 | oGM196 | 5- CCA GGA TAC ATA GAT TAC CAC AAC TCC GAG -3 |
|  | oGM347 | 5- ACG AGC AGC AAA TAT AGG TGG TGG AAG GGC TCG GAG TTG TGG TAA TCT ATG TAT CCT GG -3 |
|  | oGM76 | 5- ACC TAT ATT TGC TGC TCG TGT TTA CTC TCA CTG ACA GCT AGC TCA GTC CTA GGT ATA ATG -3 |
|  | oGM53 | 5- ACT CAT CAG ACC GGA AAG CAC ATC CGG TGA CAG CTG CTA GCA TTA TAC CTA GGA CTG AGC -3 |
|  | oGM54 | 5- TGC TTT CCG GTC TGA TGA GTC CGT GAG GAC GAA ACA GCC TCT ACA AAT AAT TTT GTT TAA -3 |
|  | oGM335 | 5- AGG TAA TTC TTT CCT TAT AAA CTA TTG GAC TTA TTA AAC AAA ATT ATT TGT AGA GGC T -3 |
|  | oGM343 | 5- GTT TAT AAG GAA AGA ATT ACC TAT GCG TAA AGG CGA AGA GCT GTT CAC TGG TGT CGT CCC -3 |
|  | oGM345 | 5- GGG ACG ACA CCA GTG AAC AGC -3 |
| J23108+UP | oGM196 | 5- CCA GGA TAC ATA GAT TAC CAC AAC TCC GAG -3 |
|  | oGM198 | 5- TAA AAA AAT TTT CCA TAG GTG GTG GAA GGG CTC GGA GTT GTG GTA ATC TAT GTA TCC TGG -3 |
|  | oGM77 | 5- ACC TAT GGA AAA TTT TTT TAA AAA AAA AAC CTG ACA GCT AGC TCA GTC CTA GGT ATA ATG -3 |
|  | oGM53 | 5- ACT CAT CAG ACC GGA AAG CAC ATC CGG TGA CAG CTG CTA GCA TTA TAC CTA GGA CTG AGC -3 |
|  | oGM54 | 5- TGC TTT CCG GTC TGA TGA GTC CGT GAG GAC GAA ACA GCC TCT ACA AAT AAT TTT GTT TAA -3 |
|  | oGM335 | 5- AGG TAA TTC TTT CCT TAT AAA CTA TTG GAC TTA TTA AAC AAA ATT ATT TGT AGA GGC T -3 |
|  | oGM343 | 5- GTT TAT AAG GAA AGA ATT ACC TAT GCG TAA AGG CGA AGA GCT GTT CAC TGG TGT CGT CCC -3 |
|  | oGM345 | 5- GGG ACG ACA CCA GTG AAC AGC -3 |
| J23106 | oGM196 | 5- CCA GGA TAC ATA GAT TAC CAC AAC TCC GAG -3 |
|  | oGM347 | 5- ACG AGC AGC AAA TAT AGG TGG TGG AAG GGC TCG GAG TTG TGG TAA TCT ATG TAT CCT GG -3 |
|  | oGM78 | 5- ACC TAT ATT TGC TGC TCG TGT TTA CTC TCA TTT ACG GCT AGC TCA GTC CTA GGT ATA GTG -3 |
|  | oGM72 | 5- ACT CAT CAG ACC GGA AAG CAC ATC CGG TGA CAG CTG CTA GCA CTA TAC CTA GGA CTG AGC -3 |
|  | oGM54 | 5- TGC TTT CCG GTC TGA TGA GTC CGT GAG GAC GAA ACA GCC TCT ACA AAT AAT TTT GTT TAA -3 |
|  | oGM335 | 5- AGG TAA TTC TTT CCT TAT AAA CTA TTG GAC TTA TTA AAC AAA ATT ATT TGT AGA GGC T -3 |
|  | oGM343 | 5- GTT TAT AAG GAA AGA ATT ACC TAT GCG TAA AGG CGA AGA GCT GTT CAC TGG TGT CGT CCC -3 |
|  | oGM345 | 5- GGG ACG ACA CCA GTG AAC AGC -3 |
| J23106+UP | oGM196 | 5- CCA GGA TAC ATA GAT TAC CAC AAC TCC GAG -3 |
|  | oGM198 | 5- TAA AAA AAT TTT CCA TAG GTG GTG GAA GGG CTC GGA GTT GTG GTA ATC TAT GTA TCC TGG -3 |
|  | oGM79 | 5- ACC TAT GGA AAA TTT TTT TAA AAA AAA AAC TTT ACG GCT AGC TCA GTC CTA GGT ATA GTG -3 |
|  | oGM72 | 5- ACT CAT CAG ACC GGA AAG CAC ATC CGG TGA CAG CTG CTA GCA CTA TAC CTA GGA CTG AGC -3 |
|  | oGM54 | 5- TGC TTT CCG GTC TGA TGA GTC CGT GAG GAC GAA ACA GCC TCT ACA AAT AAT TTT GTT TAA -3 |
|  | oGM335 | 5- AGG TAA TTC TTT CCT TAT AAA CTA TTG GAC TTA TTA AAC AAA ATT ATT TGT AGA GGC T -3 |
|  | oGM343 | 5- GTT TAT AAG GAA AGA ATT ACC TAT GCG TAA AGG CGA AGA GCT GTT CAC TGG TGT CGT CCC -3 |
|  | oGM345 | 5- GGG ACG ACA CCA GTG AAC AGC -3 |
| J23107 | oGM196 | 5- CCA GGA TAC ATA GAT TAC CAC AAC TCC GAG -3 |
|  | oGM347 | 5- ACG AGC AGC AAA TAT AGG TGG TGG AAG GGC TCG GAG TTG TGG TAA TCT ATG TAT CCT GG -3 |
|  | oGM80 | 5- ACC TAT ATT TGC TGC TCG TGT TTA CTC TCA TTT ACG GCT AGC TCA GCC CTA GGT ATT ATG -3 |
|  | oGM81 | 5- ACT CAT CAG ACC GGA AAG CAC ATC CGG TGA CAG CTG CTA GCA TAA TAC CTA GGG CTG AGC -3 |
|  | oGM54 | 5- TGC TTT CCG GTC TGA TGA GTC CGT GAG GAC GAA ACA GCC TCT ACA AAT AAT TTT GTT TAA -3 |
|  | oGM335 | 5- AGG TAA TTC TTT CCT TAT AAA CTA TTG GAC TTA TTA AAC AAA ATT ATT TGT AGA GGC T -3 |
|  | oGM343 | 5- GTT TAT AAG GAA AGA ATT ACC TAT GCG TAA AGG CGA AGA GCT GTT CAC TGG TGT CGT CCC -3 |
|  | oGM345 | 5- GGG ACG ACA CCA GTG AAC AGC -3 |
| J23107+UP | oGM196 | 5- CCA GGA TAC ATA GAT TAC CAC AAC TCC GAG -3 |
|  | oGM198 | 5- TAA AAA AAT TTT CCA TAG GTG GTG GAA GGG CTC GGA GTT GTG GTA ATC TAT GTA TCC TGG -3 |
|  | oGM82 | 5- ACC TAT GGA AAA TTT TTT TAA AAA AAA AAC TTT ACG GCT AGC TCA GCC CTA GGT ATT ATG -3 |
|  | oGM81 | 5- ACT CAT CAG ACC GGA AAG CAC ATC CGG TGA CAG CTG CTA GCA TAA TAC CTA GGG CTG AGC -3 |
|  | oGM54 | 5- TGC TTT CCG GTC TGA TGA GTC CGT GAG GAC GAA ACA GCC TCT ACA AAT AAT TTT GTT TAA -3 |
|  | oGM335 | 5- AGG TAA TTC TTT CCT TAT AAA CTA TTG GAC TTA TTA AAC AAA ATT ATT TGT AGA GGC T -3 |
|  | oGM343 | 5- GTT TAT AAG GAA AGA ATT ACC TAT GCG TAA AGG CGA AGA GCT GTT CAC TGG TGT CGT CCC -3 |
|  | oGM345 | 5- GGG ACG ACA CCA GTG AAC AGC -3 |
| J23110 | oGM196 | 5- CCA GGA TAC ATA GAT TAC CAC AAC TCC GAG -3 |
|  | oGM347 | 5- ACG AGC AGC AAA TAT AGG TGG TGG AAG GGC TCG GAG TTG TGG TAA TCT ATG TAT CCT GG -3 |
|  | oGM83 | 5- ACC TAT ATT TGC TGC TCG TGT TTA CTC TCA TTT ACG GCT AGC TCA GTC CTA GGT ACA ATG -3 |
|  | oGM84 | 5- ACT CAT CAG ACC GGA AAG CAC ATC CGG TGA CAG CTG CTA GCA TTG TAC CTA GGA CTG AGC -3 |
|  | oGM54 | 5- TGC TTT CCG GTC TGA TGA GTC CGT GAG GAC GAA ACA GCC TCT ACA AAT AAT TTT GTT TAA -3 |
|  | oGM335 | 5- AGG TAA TTC TTT CCT TAT AAA CTA TTG GAC TTA TTA AAC AAA ATT ATT TGT AGA GGC T -3 |
|  | oGM343 | 5- GTT TAT AAG GAA AGA ATT ACC TAT GCG TAA AGG CGA AGA GCT GTT CAC TGG TGT CGT CCC -3 |
|  | oGM345 | 5- GGG ACG ACA CCA GTG AAC AGC -3 |
| J23110+UP | oGM196 | 5- CCA GGA TAC ATA GAT TAC CAC AAC TCC GAG -3 |
|  | oGM198 | 5- TAA AAA AAT TTT CCA TAG GTG GTG GAA GGG CTC GGA GTT GTG GTA ATC TAT GTA TCC TGG -3 |
|  | oGM85 | 5- ACC TAT GGA AAA TTT TTT TAA AAA AAA AAC TTT ACG GCT AGC TCA GTC CTA GGT ACA ATG -3 |
|  | oGM84 | 5- ACT CAT CAG ACC GGA AAG CAC ATC CGG TGA CAG CTG CTA GCA TTG TAC CTA GGA CTG AGC -3 |
|  | oGM54 | 5- TGC TTT CCG GTC TGA TGA GTC CGT GAG GAC GAA ACA GCC TCT ACA AAT AAT TTT GTT TAA -3 |
|  | oGM335 | 5- AGG TAA TTC TTT CCT TAT AAA CTA TTG GAC TTA TTA AAC AAA ATT ATT TGT AGA GGC T -3 |
|  | oGM343 | 5- GTT TAT AAG GAA AGA ATT ACC TAT GCG TAA AGG CGA AGA GCT GTT CAC TGG TGT CGT CCC -3 |
|  | oGM345 | 5- GGG ACG ACA CCA GTG AAC AGC -3 |
| J23105 | oGM196 | 5- CCA GGA TAC ATA GAT TAC CAC AAC TCC GAG -3 |
|  | oGM347 | 5- ACG AGC AGC AAA TAT AGG TGG TGG AAG GGC TCG GAG TTG TGG TAA TCT ATG TAT CCT GG -3 |
|  | oGM86 | 5- ACC TAT ATT TGC TGC TCG TGT TTA CTC TCA TTT ACG GCT AGC TCA GTC CTA GGT ACT ATG -3 |
|  | oGM87 | 5- ACT CAT CAG ACC GGA AAG CAC ATC CGG TGA CAG CTG CTA GCA TAG TAC CTA GGA CTG AGC -3 |
|  | oGM54 | 5- TGC TTT CCG GTC TGA TGA GTC CGT GAG GAC GAA ACA GCC TCT ACA AAT AAT TTT GTT TAA -3 |
|  | oGM335 | 5- AGG TAA TTC TTT CCT TAT AAA CTA TTG GAC TTA TTA AAC AAA ATT ATT TGT AGA GGC T -3 |
|  | oGM343 | 5- GTT TAT AAG GAA AGA ATT ACC TAT GCG TAA AGG CGA AGA GCT GTT CAC TGG TGT CGT CCC -3 |
|  | oGM345 | 5- GGG ACG ACA CCA GTG AAC AGC -3 |
| J23105+UP | oGM196 | 5- CCA GGA TAC ATA GAT TAC CAC AAC TCC GAG -3 |
|  | oGM198 | 5- TAA AAA AAT TTT CCA TAG GTG GTG GAA GGG CTC GGA GTT GTG GTA ATC TAT GTA TCC TGG -3 |
|  | oGM88 | 5- ACC TAT GGA AAA TTT TTT TAA AAA AAA AAC TTT ACG GCT AGC TCA GTC CTA GGT ACT ATG -3 |
|  | oGM87 | 5- ACT CAT CAG ACC GGA AAG CAC ATC CGG TGA CAG CTG CTA GCA TAG TAC CTA GGA CTG AGC -3 |
|  | oGM54 | 5- TGC TTT CCG GTC TGA TGA GTC CGT GAG GAC GAA ACA GCC TCT ACA AAT AAT TTT GTT TAA -3 |
|  | oGM335 | 5- AGG TAA TTC TTT CCT TAT AAA CTA TTG GAC TTA TTA AAC AAA ATT ATT TGT AGA GGC T -3 |
|  | oGM343 | 5- GTT TAT AAG GAA AGA ATT ACC TAT GCG TAA AGG CGA AGA GCT GTT CAC TGG TGT CGT CCC -3 |
|  | oGM345 | 5- GGG ACG ACA CCA GTG AAC AGC -3 |
| J23116 | oGM196 | 5- CCA GGA TAC ATA GAT TAC CAC AAC TCC GAG -3 |
|  | oGM347 | 5- ACG AGC AGC AAA TAT AGG TGG TGG AAG GGC TCG GAG TTG TGG TAA TCT ATG TAT CCT GG -3 |
|  | oGM89 | 5- ACC TAT ATT TGC TGC TCG TGT TTA CTC TCA TTG ACA GCT AGC TCA GTC CTA GGG ACT ATG -3 |
|  | oGM90 | 5- ACT CAT CAG ACC GGA AAG CAC ATC CGG TGA CAG CTG CTA GCA TAG TCC CTA GGA CTG AGC -3 |
|  | oGM54 | 5- TGC TTT CCG GTC TGA TGA GTC CGT GAG GAC GAA ACA GCC TCT ACA AAT AAT TTT GTT TAA -3 |
|  | oGM335 | 5- AGG TAA TTC TTT CCT TAT AAA CTA TTG GAC TTA TTA AAC AAA ATT ATT TGT AGA GGC T -3 |
|  | oGM343 | 5- GTT TAT AAG GAA AGA ATT ACC TAT GCG TAA AGG CGA AGA GCT GTT CAC TGG TGT CGT CCC -3 |
|  | oGM345 | 5- GGG ACG ACA CCA GTG AAC AGC -3 |
| J23116+UP | oGM196 | 5- CCA GGA TAC ATA GAT TAC CAC AAC TCC GAG -3 |
|  | oGM198 | 5- TAA AAA AAT TTT CCA TAG GTG GTG GAA GGG CTC GGA GTT GTG GTA ATC TAT GTA TCC TGG -3 |
|  | oGM91 | 5- ACC TAT GGA AAA TTT TTT TAA AAA AAA AAC TTG ACA GCT AGC TCA GTC CTA GGG ACT ATG -3 |
|  | oGM90 | 5- ACT CAT CAG ACC GGA AAG CAC ATC CGG TGA CAG CTG CTA GCA TAG TCC CTA GGA CTG AGC -3 |
|  | oGM54 | 5- TGC TTT CCG GTC TGA TGA GTC CGT GAG GAC GAA ACA GCC TCT ACA AAT AAT TTT GTT TAA -3 |
|  | oGM335 | 5- AGG TAA TTC TTT CCT TAT AAA CTA TTG GAC TTA TTA AAC AAA ATT ATT TGT AGA GGC T -3 |
|  | oGM343 | 5- GTT TAT AAG GAA AGA ATT ACC TAT GCG TAA AGG CGA AGA GCT GTT CAC TGG TGT CGT CCC -3 |
|  | oGM345 | 5- GGG ACG ACA CCA GTG AAC AGC -3 |
| J23115 | oGM196 | 5- CCA GGA TAC ATA GAT TAC CAC AAC TCC GAG -3 |
|  | oGM347 | 5- ACG AGC AGC AAA TAT AGG TGG TGG AAG GGC TCG GAG TTG TGG TAA TCT ATG TAT CCT GG -3 |
|  | oGM92 | 5- ACC TAT ATT TGC TGC TCG TGT TTA CTC TCA TTT ATA GCT AGC TCA GCC CTT GGT ACA ATG -3 |
|  | oGM93 | 5- ACT CAT CAG ACC GGA AAG CAC ATC CGG TGA CAG CTG CTA GCA TTG TAC CAA GGG CTG AGC -3 |
|  | oGM54 | 5- TGC TTT CCG GTC TGA TGA GTC CGT GAG GAC GAA ACA GCC TCT ACA AAT AAT TTT GTT TAA -3 |
|  | oGM335 | 5- AGG TAA TTC TTT CCT TAT AAA CTA TTG GAC TTA TTA AAC AAA ATT ATT TGT AGA GGC T -3 |
|  | oGM343 | 5- GTT TAT AAG GAA AGA ATT ACC TAT GCG TAA AGG CGA AGA GCT GTT CAC TGG TGT CGT CCC -3 |
|  | oGM345 | 5- GGG ACG ACA CCA GTG AAC AGC -3 |
| J23115+UP | oGM196 | 5- CCA GGA TAC ATA GAT TAC CAC AAC TCC GAG -3 |
|  | oGM198 | 5- TAA AAA AAT TTT CCA TAG GTG GTG GAA GGG CTC GGA GTT GTG GTA ATC TAT GTA TCC TGG -3 |
|  | oGM94 | 5- ACC TAT GGA AAA TTT TTT TAA AAA AAA AAC TTT ATA GCT AGC TCA GCC CTT GGT ACA ATG -3 |
|  | oGM93 | 5- ACT CAT CAG ACC GGA AAG CAC ATC CGG TGA CAG CTG CTA GCA TTG TAC CAA GGG CTG AGC -3 |
|  | oGM54 | 5- TGC TTT CCG GTC TGA TGA GTC CGT GAG GAC GAA ACA GCC TCT ACA AAT AAT TTT GTT TAA -3 |
|  | oGM335 | 5- AGG TAA TTC TTT CCT TAT AAA CTA TTG GAC TTA TTA AAC AAA ATT ATT TGT AGA GGC T -3 |
|  | oGM343 | 5- GTT TAT AAG GAA AGA ATT ACC TAT GCG TAA AGG CGA AGA GCT GTT CAC TGG TGT CGT CCC -3 |
|  | oGM345 | 5- GGG ACG ACA CCA GTG AAC AGC -3 |
| J23114 | oGM196 | 5- CCA GGA TAC ATA GAT TAC CAC AAC TCC GAG -3 |
|  | oGM347 | 5- ACG AGC AGC AAA TAT AGG TGG TGG AAG GGC TCG GAG TTG TGG TAA TCT ATG TAT CCT GG -3 |
|  | oGM95 | 5- ACC TAT ATT TGC TGC TCG TGT TTA CTC TCA TTT ATG GCT AGC TCA GTC CTA GGT ACA ATG -3 |
|  | oGM84 | 5- ACT CAT CAG ACC GGA AAG CAC ATC CGG TGA CAG CTG CTA GCA TTG TAC CTA GGA CTG AGC -3 |
|  | oGM54 | 5- TGC TTT CCG GTC TGA TGA GTC CGT GAG GAC GAA ACA GCC TCT ACA AAT AAT TTT GTT TAA -3 |
|  | oGM335 | 5- AGG TAA TTC TTT CCT TAT AAA CTA TTG GAC TTA TTA AAC AAA ATT ATT TGT AGA GGC T -3 |
|  | oGM343 | 5- GTT TAT AAG GAA AGA ATT ACC TAT GCG TAA AGG CGA AGA GCT GTT CAC TGG TGT CGT CCC -3 |
|  | oGM345 | 5- GGG ACG ACA CCA GTG AAC AGC -3 |
| J23114+UP | oGM196 | 5- CCA GGA TAC ATA GAT TAC CAC AAC TCC GAG -3 |
|  | oGM198 | 5- TAA AAA AAT TTT CCA TAG GTG GTG GAA GGG CTC GGA GTT GTG GTA ATC TAT GTA TCC TGG -3 |
|  | oGM96 | 5- ACC TAT GGA AAA TTT TTT TAA AAA AAA AAC TTT ATG GCT AGC TCA GTC CTA GGT ACA ATG -3 |
|  | oGM84 | 5- ACT CAT CAG ACC GGA AAG CAC ATC CGG TGA CAG CTG CTA GCA TTG TAC CTA GGA CTG AGC -3 |
|  | oGM54 | 5- TGC TTT CCG GTC TGA TGA GTC CGT GAG GAC GAA ACA GCC TCT ACA AAT AAT TTT GTT TAA -3 |
|  | oGM335 | 5- AGG TAA TTC TTT CCT TAT AAA CTA TTG GAC TTA TTA AAC AAA ATT ATT TGT AGA GGC T -3 |
|  | oGM343 | 5- GTT TAT AAG GAA AGA ATT ACC TAT GCG TAA AGG CGA AGA GCT GTT CAC TGG TGT CGT CCC -3 |
|  | oGM345 | 5- GGG ACG ACA CCA GTG AAC AGC -3 |
| J23117 | oGM196 | 5- CCA GGA TAC ATA GAT TAC CAC AAC TCC GAG -3 |
|  | oGM347 | 5- ACG AGC AGC AAA TAT AGG TGG TGG AAG GGC TCG GAG TTG TGG TAA TCT ATG TAT CCT GG -3 |
|  | oGM97 | 5- ACC TAT ATT TGC TGC TCG TGT TTA CTC TCA TTG ACA GCT AGC TCA GTC CTA GGG ATT GTG -3 |
|  | oGM98 | 5- ACT CAT CAG ACC GGA AAG CAC ATC CGG TGA CAG CTG CTA GCA CAA TCC CTA GGA CTG AGC -3 |
|  | oGM54 | 5- TGC TTT CCG GTC TGA TGA GTC CGT GAG GAC GAA ACA GCC TCT ACA AAT AAT TTT GTT TAA -3 |
|  | oGM335 | 5- AGG TAA TTC TTT CCT TAT AAA CTA TTG GAC TTA TTA AAC AAA ATT ATT TGT AGA GGC T -3 |
|  | oGM343 | 5- GTT TAT AAG GAA AGA ATT ACC TAT GCG TAA AGG CGA AGA GCT GTT CAC TGG TGT CGT CCC -3 |
|  | oGM345 | 5- GGG ACG ACA CCA GTG AAC AGC -3 |
| J23117+UP | oGM196 | 5- CCA GGA TAC ATA GAT TAC CAC AAC TCC GAG -3 |
|  | oGM198 | 5- TAA AAA AAT TTT CCA TAG GTG GTG GAA GGG CTC GGA GTT GTG GTA ATC TAT GTA TCC TGG -3 |
|  | oGM99 | 5- ACC TAT GGA AAA TTT TTT TAA AAA AAA AAC TTG ACA GCT AGC TCA GTC CTA GGG ATT GTG -3 |
|  | oGM98 | 5- ACT CAT CAG ACC GGA AAG CAC ATC CGG TGA CAG CTG CTA GCA CAA TCC CTA GGA CTG AGC -3 |
|  | oGM54 | 5- TGC TTT CCG GTC TGA TGA GTC CGT GAG GAC GAA ACA GCC TCT ACA AAT AAT TTT GTT TAA -3 |
|  | oGM335 | 5- AGG TAA TTC TTT CCT TAT AAA CTA TTG GAC TTA TTA AAC AAA ATT ATT TGT AGA GGC T -3 |
|  | oGM343 | 5- GTT TAT AAG GAA AGA ATT ACC TAT GCG TAA AGG CGA AGA GCT GTT CAC TGG TGT CGT CCC -3 |
|  | oGM345 | 5- GGG ACG ACA CCA GTG AAC AGC -3 |
| J23109 | oGM196 | 5- CCA GGA TAC ATA GAT TAC CAC AAC TCC GAG -3 |
|  | oGM347 | 5- ACG AGC AGC AAA TAT AGG TGG TGG AAG GGC TCG GAG TTG TGG TAA TCT ATG TAT CCT GG -3 |
|  | oGM100 | 5- ACC TAT ATT TGC TGC TCG TGT TTA CTC TCA TTT ACA GCT AGC TCA GTC CTA GGG ACT GTG -3 |
|  | oGM101 | 5- ACT CAT CAG ACC GGA AAG CAC ATC CGG TGA CAG CTG CTA GCA CAG TCC CTA GGA CTG AGC -3 |
|  | oGM54 | 5- TGC TTT CCG GTC TGA TGA GTC CGT GAG GAC GAA ACA GCC TCT ACA AAT AAT TTT GTT TAA -3 |
|  | oGM335 | 5- AGG TAA TTC TTT CCT TAT AAA CTA TTG GAC TTA TTA AAC AAA ATT ATT TGT AGA GGC T -3 |
|  | oGM343 | 5- GTT TAT AAG GAA AGA ATT ACC TAT GCG TAA AGG CGA AGA GCT GTT CAC TGG TGT CGT CCC -3 |
|  | oGM345 | 5- GGG ACG ACA CCA GTG AAC AGC -3 |
| J23109+UP | oGM196 | 5- CCA GGA TAC ATA GAT TAC CAC AAC TCC GAG -3 |
|  | oGM198 | 5- TAA AAA AAT TTT CCA TAG GTG GTG GAA GGG CTC GGA GTT GTG GTA ATC TAT GTA TCC TGG -3 |
|  | oGM102 | 5- ACC TAT GGA AAA TTT TTT TAA AAA AAA AAC TTT ACA GCT AGC TCA GTC CTA GGG ACT GTG -3 |
|  | oGM101 | 5- ACT CAT CAG ACC GGA AAG CAC ATC CGG TGA CAG CTG CTA GCA CAG TCC CTA GGA CTG AGC -3 |
|  | oGM54 | 5- TGC TTT CCG GTC TGA TGA GTC CGT GAG GAC GAA ACA GCC TCT ACA AAT AAT TTT GTT TAA -3 |
|  | oGM335 | 5- AGG TAA TTC TTT CCT TAT AAA CTA TTG GAC TTA TTA AAC AAA ATT ATT TGT AGA GGC T -3 |
|  | oGM343 | 5- GTT TAT AAG GAA AGA ATT ACC TAT GCG TAA AGG CGA AGA GCT GTT CAC TGG TGT CGT CCC -3 |
|  | oGM345 | 5- GGG ACG ACA CCA GTG AAC AGC -3 |
| J23113 | oGM196 | 5- CCA GGA TAC ATA GAT TAC CAC AAC TCC GAG -3 |
|  | oGM347 | 5- ACG AGC AGC AAA TAT AGG TGG TGG AAG GGC TCG GAG TTG TGG TAA TCT ATG TAT CCT GG -3 |
|  | oGM103 | 5- ACC TAT ATT TGC TGC TCG TGT TTA CTC TCA CTG ATG GCT AGC TCA GTC CTA GGG ATT ATG -3 |
|  | oGM104 | 5- ACT CAT CAG ACC GGA AAG CAC ATC CGG TGA CAG CTG CTA GCA TAA TCC CTA GGA CTG AGC -3 |
|  | oGM54 | 5- TGC TTT CCG GTC TGA TGA GTC CGT GAG GAC GAA ACA GCC TCT ACA AAT AAT TTT GTT TAA -3 |
|  | oGM335 | 5- AGG TAA TTC TTT CCT TAT AAA CTA TTG GAC TTA TTA AAC AAA ATT ATT TGT AGA GGC T -3 |
|  | oGM343 | 5- GTT TAT AAG GAA AGA ATT ACC TAT GCG TAA AGG CGA AGA GCT GTT CAC TGG TGT CGT CCC -3 |
|  | oGM345 | 5- GGG ACG ACA CCA GTG AAC AGC -3 |
| J23113+UP | oGM196 | 5- CCA GGA TAC ATA GAT TAC CAC AAC TCC GAG -3 |
|  | oGM198 | 5- TAA AAA AAT TTT CCA TAG GTG GTG GAA GGG CTC GGA GTT GTG GTA ATC TAT GTA TCC TGG -3 |
|  | oGM105 | 5- ACC TAT GGA AAA TTT TTT TAA AAA AAA AAC CTG ATG GCT AGC TCA GTC CTA GGG ATT ATG -3 |
|  | oGM104 | 5- ACT CAT CAG ACC GGA AAG CAC ATC CGG TGA CAG CTG CTA GCA TAA TCC CTA GGA CTG AGC -3 |
|  | oGM54 | 5- TGC TTT CCG GTC TGA TGA GTC CGT GAG GAC GAA ACA GCC TCT ACA AAT AAT TTT GTT TAA -3 |
|  | oGM335 | 5- AGG TAA TTC TTT CCT TAT AAA CTA TTG GAC TTA TTA AAC AAA ATT ATT TGT AGA GGC T -3 |
|  | oGM343 | 5- GTT TAT AAG GAA AGA ATT ACC TAT GCG TAA AGG CGA AGA GCT GTT CAC TGG TGT CGT CCC -3 |
|  | oGM345 | 5- GGG ACG ACA CCA GTG AAC AGC -3 |
| J23112/J23103 | oGM196 | 5- CCA GGA TAC ATA GAT TAC CAC AAC TCC GAG -3 |
|  | oGM347 | 5- ACG AGC AGC AAA TAT AGG TGG TGG AAG GGC TCG GAG TTG TGG TAA TCT ATG TAT CCT GG -3 |
|  | oGM106 | 5- ACC TAT ATT TGC TGC TCG TGT TTA CTC TCA CTG ATA GCT AGC TCA GTC CTA GGG ATT ATG -3 |
|  | oGM104 | 5- ACT CAT CAG ACC GGA AAG CAC ATC CGG TGA CAG CTG CTA GCA TAA TCC CTA GGA CTG AGC -3 |
|  | oGM54 | 5- TGC TTT CCG GTC TGA TGA GTC CGT GAG GAC GAA ACA GCC TCT ACA AAT AAT TTT GTT TAA -3 |
|  | oGM335 | 5- AGG TAA TTC TTT CCT TAT AAA CTA TTG GAC TTA TTA AAC AAA ATT ATT TGT AGA GGC T -3 |
|  | oGM343 | 5- GTT TAT AAG GAA AGA ATT ACC TAT GCG TAA AGG CGA AGA GCT GTT CAC TGG TGT CGT CCC -3 |
|  | oGM345 | 5- GGG ACG ACA CCA GTG AAC AGC -3 |
| J23112/J23103+UP | oGM196 | 5- CCA GGA TAC ATA GAT TAC CAC AAC TCC GAG -3 |
|  | oGM198 | 5- TAA AAA AAT TTT CCA TAG GTG GTG GAA GGG CTC GGA GTT GTG GTA ATC TAT GTA TCC TGG -3 |
|  | oGM107 | 5- ACC TAT GGA AAA TTT TTT TAA AAA AAA AAC CTG ATA GCT AGC TCA GTC CTA GGG ATT ATG -3 |
|  | oGM104 | 5- ACT CAT CAG ACC GGA AAG CAC ATC CGG TGA CAG CTG CTA GCA TAA TCC CTA GGA CTG AGC -3 |
|  | oGM54 | 5- TGC TTT CCG GTC TGA TGA GTC CGT GAG GAC GAA ACA GCC TCT ACA AAT AAT TTT GTT TAA -3 |
|  | oGM335 | 5- AGG TAA TTC TTT CCT TAT AAA CTA TTG GAC TTA TTA AAC AAA ATT ATT TGT AGA GGC T -3 |
|  | oGM343 | 5- GTT TAT AAG GAA AGA ATT ACC TAT GCG TAA AGG CGA AGA GCT GTT CAC TGG TGT CGT CCC -3 |
|  | oGM345 | 5- GGG ACG ACA CCA GTG AAC AGC -3 |

**Table S2**. Primers and DNA sequences used in this study.

| primer name | primer sequences (5'-3') |
| --- | --- |
| J23119 1/2UP-f | CCAGGATACATAGATTACCACAACTCCGAGcccttccaccacctatatttgctgctcgtAAAAAAAAAACTTGACAGCTAGCTCAG^a^ |
| J23108 1/2UP-f | CCAGGATACATAGATTACCACAACTCCGAGCCCTTCCACCACCTATATTTGCTGCTCGTAAAAAAAAAACCTGACAGCTAGC |
| J23115 1/2UP-f | CCAGGATACATAGATTACCACAACTCCGAGCCCTTCCACCACCTATATTTGCTGCTCGTAAAAAAAAAACTTTATAGCTAGCTCAGC |
| J23109 1/2UP-f | CCAGGATACATAGATTACCACAACTCCGAGCCCTTCCACCACCTATATTTGCTGCTCGTAAAAAAAAAACTTTACAGCTAGCTCA |
| J23112 1/2UP-f | CCAGGATACATAGATTACCACAACTCCGAGCCCTTCCACCACCTATATTTGCTGCTCGTAAAAAAAAAACCTGATAGCTAGCTC |
| J23102 1/2UP-f | CCAGGATACATAGATTACCACAACTCCGAGCCCTTCCACCACCTATATTTGCTGCTCGTAAAAAAAAAACTTGACAGCTAGCT |
| UNSx-f | CCAGGATACATAGATTACCACAAC |
| UNS5-f | GAGCCAACTCCCTTTACAAC |
| GFP-r | GGGACGACACCAGTGAA |
| **Gblocks** | **Sequence (5'-3')** |
| GFP-UNS5 | atgcgtaaaggcgaagagctgttcactggtgtcgtccctattctggtggaactggatggtgatgtcaacggtcataagttttccgtgcgtggcgagggtgaaggtgacgcaactaatggtaaactgacgctgaagttcatctgtactactggtaaactgccggtaccttggccgactctggtaacgacgctgacttatggtgttcagtgctttgctcgttatccggaccatatgaagcagcatgacttcttcaagtccgccatgccggaaggctatgtgcaggaacgcacgatttcctttaaggatgacggcacgtacaaaacgcgtgcggaagtgaaatttgaaggcgataccctggtaaaccgcattgagctgaaaggcattgactttaaagaagacggcaatatcctgggccataagctggaatacaattttaacagccacaatgtttacatcaccgccgataaacaaaaaaatggcattaaagcgaattttaaaattcgccacaacgtggaggatggcagcgtgcagctggctgatcactaccagcaaaacactccaatcggtgatggtcctgttctgctgccagacaatcactatctgagcacgcaaagcgttctgtctaaagatccgaacgagaaacgcgatcatatggttctgctggagttcgtaaccgcagcgggcatcacgcatggtatggatgaactgtacaaatgatgaCTCTAACGGACTTGAGTGAGGTTGTAAAGGGAGTTGGCTC |

^a^Red color represents overlap portion of the primer that anneals at template DN

**
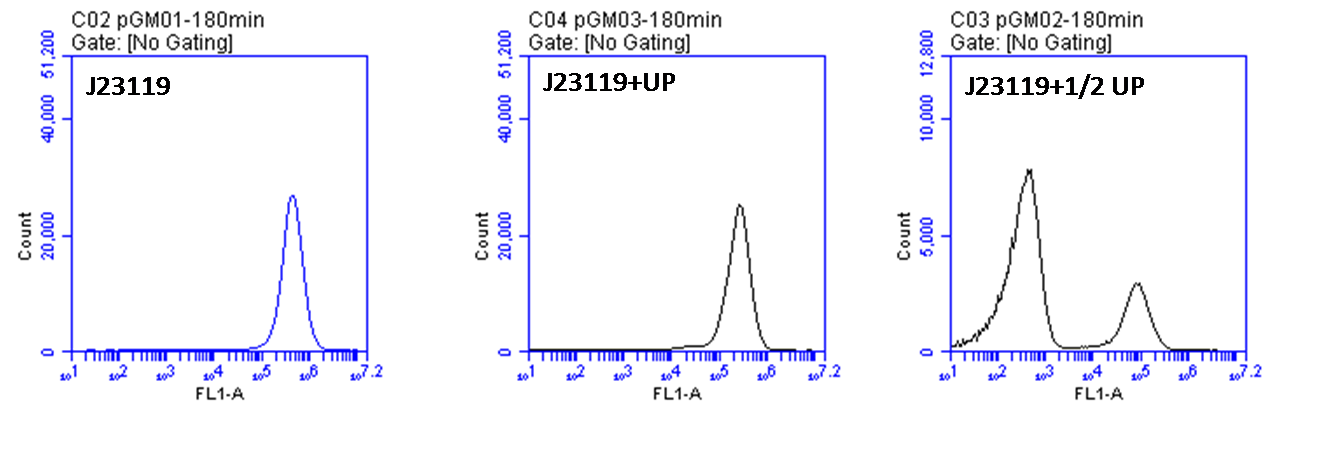
**

**(A) (B) (C)**

**Fig. S3.** Representatives of fluorescence measurement results. (A) J23119; (B) J23119 with full UP element; (C) J23119 with half UP element. For each figure, X-axis is fluorescence intensity and Y-axis is cell-counting number. Gene expression was obtained as the distribution of fluorescence intensity of each single cell to the overall cell population (CV). All fluorescence results were obtained by harvesting cells after 2.5 h (see Methods).

**
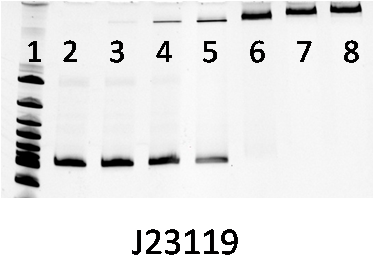

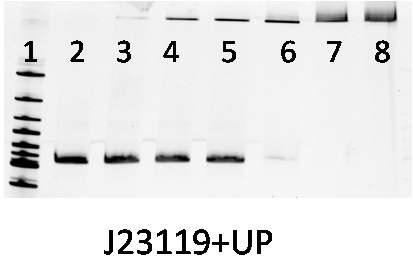

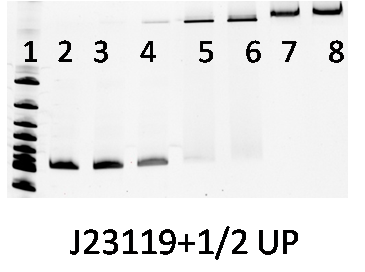
**

1. **(B) (C)**

**Fig. S4.** Representatives of EMSA experiment results. (A) J23119; (B) J23119 with full UP element; (C) J23119 with half UP element. For each figure, from lane 1 to 8 are 100 bp DNA ladder, 0 nM RNAP, 10 nM RNAP, 21 nM RNAP, 42 nM RNAP, 85 nM RNAP, 170 nM RNAP, and 340 nM RNAP. In each figure, The top row bands were binding DNA by RNAP and the bottom row bands were free DNA.
